# A Novel Risk Predictive Scoring Model for Predicting Subsequent Infection After Carbapenem-Resistant Gram-Negative Bacteria Colonization in Hematological Malignancy Patients

**DOI:** 10.3389/fonc.2022.897479

**Published:** 2022-05-11

**Authors:** Qiuling Wu, Chenjing Qian, Hua Yin, Fang Liu, Yaohui Wu, Weiming Li, Linghui Xia, Ling Ma, Mei Hong

**Affiliations:** ^1^ Institute of Hematology, Union Hospital, Tongji Medical College, Huazhong University of Science and Technology, Wuhan, China; ^2^ Department of Clinical Laboratory, Union Hospital, Tongji Medical College, Huazhong University of Science and Technology, Wuhan, China; ^3^ Collaborative Innovation Center of Hematology, Soochow University, Suzhou, China

**Keywords:** carbapenem-resistant Gram-negative bacteria, hematologic malignancies, colonization, infection, rectoanal swabs, predictive model

## Abstract

**Background:**

This study investigated the high-risk factors associated with the increased vulnerability for subsequent clinical CR-GNB infection in carbapenem-resistant Gram-negative bacteria (CR-GNB)-colonized hematological malignancy (HM) patients and built a statistical model to predict subsequent infection.

**Method:**

All adult HM patients with positive rectoanal swabs culture for CR-GNB between January 2018 and June 2020 were prospectively followed to assess for any subsequent CR-GNB infections and to investigate the risk factors and clinical features of subsequent infection.

**Results:**

A total of 392 HM patients were enrolled. Of them, 46.7% developed a subsequent clinical CR-GNB infection, with 42 (10.7%) cases of confirmed infection and 141 (36%) cases of clinically diagnosed infection. *Klebsiella pneumoniae* was the dominant species. The overall mortality rate of patients colonized and infected with CR-GNB was 8.6% and 43.7%. A multivariate analysis showed that remission induction chemotherapy and the duration of agranulocytosis, mucositis, and hypoalbuminemia were significant predictors of subsequent infection after CR-GNB colonization. According to our novel risk-predictive scoring model, the high-risk group were >3 times more likely to develop a subsequent infection in comparison with the low-risk group.

**Conclusion:**

Our risk-predictive scoring model can early and accurately predict a subsequent CR-GNB infection in HM patients with CR-GNB colonization. The early administration of CR-GNB-targeted empirical therapy in the high-risk group is strongly recommended to decrease their mortality.

## Introduction

*Carbapenem-resistant Gram-negative bacteria* (CR-GNB) are a major public health threat posed by the Centers for Disease Control and the World Health Organization (1, 2), *Carbapenem-resistant Enterobacterales* (CRE), *Carbapenem-resistant Pseudomonas aeruginosa* (CRPA), and *Carbapenem-resistant Acinetobacter baumannii* (CRA) are considered as *carbapenem-resistant organisms* (CROs) (3). In recent decades, their prevalence has increased year by year (4). The disease progresses rapidly, and the all-cause mortality of CR-GNB infected patients is high (ranging from 20% to 71%), which presents a tremendous challenge to clinicians. Limited treatment options are currently available, including ceftazidime/avibactam, aztreonam, fosfomycin, and polymyxin (5, 6). However, the delayed administration of these antibiotics still resulted in rapidly fatal outcomes.

Immunodeficiency due to primary diseases, neutropenia, high-dose chemotherapy, hematopoietic stem cell transplantation (HSCT), and the abuse of broad-spectrum antibiotics all lead to an increased risk of a CR-GNB infection in hematological malignancy (HM) patients, and the mortality rate of infection is significantly higher than that in other patients (7). More than half of HM patients are reported to die within 1 week after a CR-GNB infection, and their 30-day mortality rate is as high as 70.3% (8). Therefore, it has an extremely important clinical value to quantify the risk factors of a CR-GNB infection to guide the early diagnosis and appropriate target treatment in HM patients.

A pathogenic microorganism culture is the gold standard for a definite diagnosis of a CR-GNB infection, but a low positive rate of culture and long culture time directly delay the timing of medication and thus limit its therapeutic effect (9). Previous studies have shown that a CR-GNB infection is usually caused by colonized bacteria invading the body and colonization can early predict the existence of infection (10, 11). At present, the common methods for detecting CR-GNB colonization include a rectoanal swab culture, fecal culture, and pharyngeal wipe culture. Of them, the rectoanal swab culture is most widely adopted because the specimen is easily obtained without the contamination of miscellaneous bacteria and can accurately reflect the gastrointestinal bacterial status of the patient (12). Active surveillance of CR-GNB colonization has been demonstrated to help provide multimodal prevention and control intervention strategies and thus effectively control CR-GNB outbreaks (13). However, gut colonization patients are a heterogeneous population, with only a small proportion developing into a clinical infection. The administration of anti-CR-GNB target antibiotics only depending on CR-GNB colonization will result in the abuse of antibiotics and increased bacterial resistance. Therefore, identifying the factors that influence asymptomatic colonization to develop into a subsequent CR-GNB infection and establishing an accurate and convenient prediction model for early recognizing high-risk CR-GNB infected patients after colonization may improve the empiric antibiotic prescription and decrease the mortality rate and healthcare costs.

In this study, we compared the clinical characteristics between CR-GNB colonization patients and CR-GNB infection patients developed from CR-GNB colonization and tried to determine the high-risk factor of subsequent infection. Meanwhile, we sought to develop a novel predictive model to predict who among CR-GNB colonization patients is prone to have a subsequent clinical CR-GNB infection. This might be helpful for identifying the real patients who may benefit from the early application of an anti-CR-GNB-targeted regimen.

## Patients and Methods

### Study Design and Subjects

This study retrospectively reviewed the data of inpatients with HMs from January 2018 to June 2020 in Wuhan Union Hospital, Tongji Medical College, Huazhong University of Science and Technology. All hospitalized patients were screened for CR-GNB colonization on admission and weekly thereafter. Once positive CR-GNB screening was found, the patients were included in the study and followed up to assess subsequent infection until the patients were discharged from the hospital. Meanwhile, the clinical and microbiological data were collected. Duplicate infection from the same patient was identified as one infection, and the first culture-positive strain was recorded.

### Data Collection

Demographic and clinical data, including gender, age, primary disease, the length of hospitalization, HSCT, pneumonia, the duration of agranulocytosis, hypoalbuminemia, mucositis, exposure to antimicrobial agents or special drugs [chemotherapy, immunosuppressant, glucocorticoid, proton pump inhibitors (PPIs)] before infection, invasive devices [central venous catheter (CVC), mechanical ventilation, sputum suction, bladder catheterization] before infection, and the survival status within 28 days after we acquired the first positive rectoanal swab culture for CR-GNB were collected.

### Bacterial Identification and Drug Sensitivity Test

Once the patients demonstrated clinical infection symptoms, the biological samples including blood, rectoanal swabs, vein catheter samples, tracheal secretions, bronchoalveolar lavage fluid, intraperitoneal fluid, or pleural drainage fluid were collected for the microorganism culture according to the location of infection. Bacterial identification and drug sensitivity tests were performed using a Vitek^®^ 2 automated system (France Biomerieux) and matrix-assisted laser-desorption ionization time-of-flight mass spectrometry (Bruker Daltonics Inc., Billerica, MA, USA). The disk diffusion method (K-B method) was used to determine minimal inhibitory concentrations (MICs). All antibiotics, except tigecycline and colistin, were interpreted according to the standard of the CLSI document (14). For tigecycline and colistin, the European Committee on Antimicrobial Susceptibility Testing (EUCAST) breakpoint was used (15). *Enterobacteriaceae* with an MIC ≥4 µg/ml were considered as resistance to carbapenem. *Pseudomonas aeruginosa* and *Acinetobacter* spp. with an MIC ≥8 µg/ml were considered as resistance to carbapenem.

### Grouping

All CR-GNB-colonized patients were followed up to discharge and grouped based on clinical infection manifestation, microorganism evidence, and therapeutic reaction to antibiotics to determine any subsequent CR-GNB infections.

#### CR-GNB Colonization Group

The patients met one of the following criteria during the follow-up period:

① Non-infection: The patients had no fever and any clinical presentations of infection.
② Non-CR-GNB infection: The patients developed fever (temperature >38° C at three different times within a 12 h period or as a temperature >38.5° C in a single measurement) and/or clinical presentations of infection (16, 17). Meanwhile, the patients had a good response to antibiotics targeting non-CR-GNB (including third- and/or fourth-generation cephalosporin, carbapenem, aminoglycoside, quinolones, and vancomycin) and/or anti-fungal treatment no matter if they had positive cultures for non-CR-GNB microorganisms and fungals.

#### CR-GNB Infection Group

The patients subsequently developed fever and/or clinical presentations of infection and met one of the following criteria:

① Confirmed CR-GNB infections: Patients had the presence of CR-GNB microbiologically documented infection [the isolation of CR-GNB from blood cultures or from a well-defined site of infection (urine, respiratory secretions obtained using sterile procedures, or fluid collection) (17)].
② Probable CR-GNB infection: Patients had the absence of CR-GNB and non-CR-GNB microorganism evidence and fungal infection evidence. Conventional anti-infective treatments targeting all non-CR-GNBs and fungals were ineffective, and subsequent antibiotics targeting CR-GNB were effective.

#### Unidentified Group

The patients subsequently developed fever and/or clinical presentations of infection and met one of the following criteria. The patients in this group were finally excluded from our study.

① The patients had the absence of CR-GNB and non-CR-GNB microorganism evidence and fungal infection evidence, but the anti-infective treatments targeting all non-CR-GNB and CR-GNB were ineffective.
② The patients had confirmed non-CR-GNB or fungal infection, but non-CR-GNB targeted anti-infective treatments and anti-fungal treatments were ineffective (18).

### Related Definition

Hypoproteinemia refers to the serum albumin <30 mg/L.

Immunosuppressant therapy was defined as the use of at least 1 of the following drugs within 30 days before a CR-GNB infection, including cyclosporine, tacrolimus, and antithymocyte globulin (ATG)/antilymphocyte globulin (ALG).

Glucocorticoid refers to the use of dexamethasone within 1 month before CR-GNB infection (dose ≥20 mg/d, duration ≥5 days).

Antimicrobial exposure was defined as the use of antibiotics for more than 72 h before CR-GNB infection.

### Statistical Analysis

Categorical data were analyzed utilizing Pearson’s chi-square or Fisher’s exact test, and continuous data were analyzed utilizing the Mann–Whitney U test or Student’s t-test, as appropriate. Logistic regression (backward LR) methods (univariate, multivariate) were used to determine the infection risk factors for HM patients with CR-GNB colonization. Odds ratios (ORs) and their corresponding 95% confidence interval (CI) were calculated. The final model was constructed based on a forward stepwise method with the likelihood ratio test. To develop the risk score, variables that had statistical significance in the multivariate regression model were assigned a reference value (Wij) according to the regression coefficients (*βi*). The risk factors in this study were all categorical variables. Dummy variables were set for categories, coding “0” and “1,” and “0” (the base category) was taken as the referent risk factor profile (*WiREF*). How far each risk factor is from the base category in terms of regression units (D) was calculated. The formula was D=βi * (Wij - WiREF). The constant in the points system (B) for scoring one point was set as the minimum of regression coefficient (*βi*). Finally, the points associated with each category of each risk factor were calculated by the following: Points=D/B=βi * (Wij - WiREF)/B. The final calculation results were rounded to an integer to obtain the scores corresponding to each risk factor. The sum of the scores generated by the calculated risk factors is the predicted score for that patient. The discrimination of the model was assessed by the receiver-operator curve (ROC) characteristics and the area under the curve (AUC). An optimal breakpoint was assigned using the Youden index, and integer up. *R 3.6.1* software was used for the analyses. Statistical significance was assigned to a *P*-value of less than 0.05.

## Results

### Microbiological Characteristics

**Figure 1:**
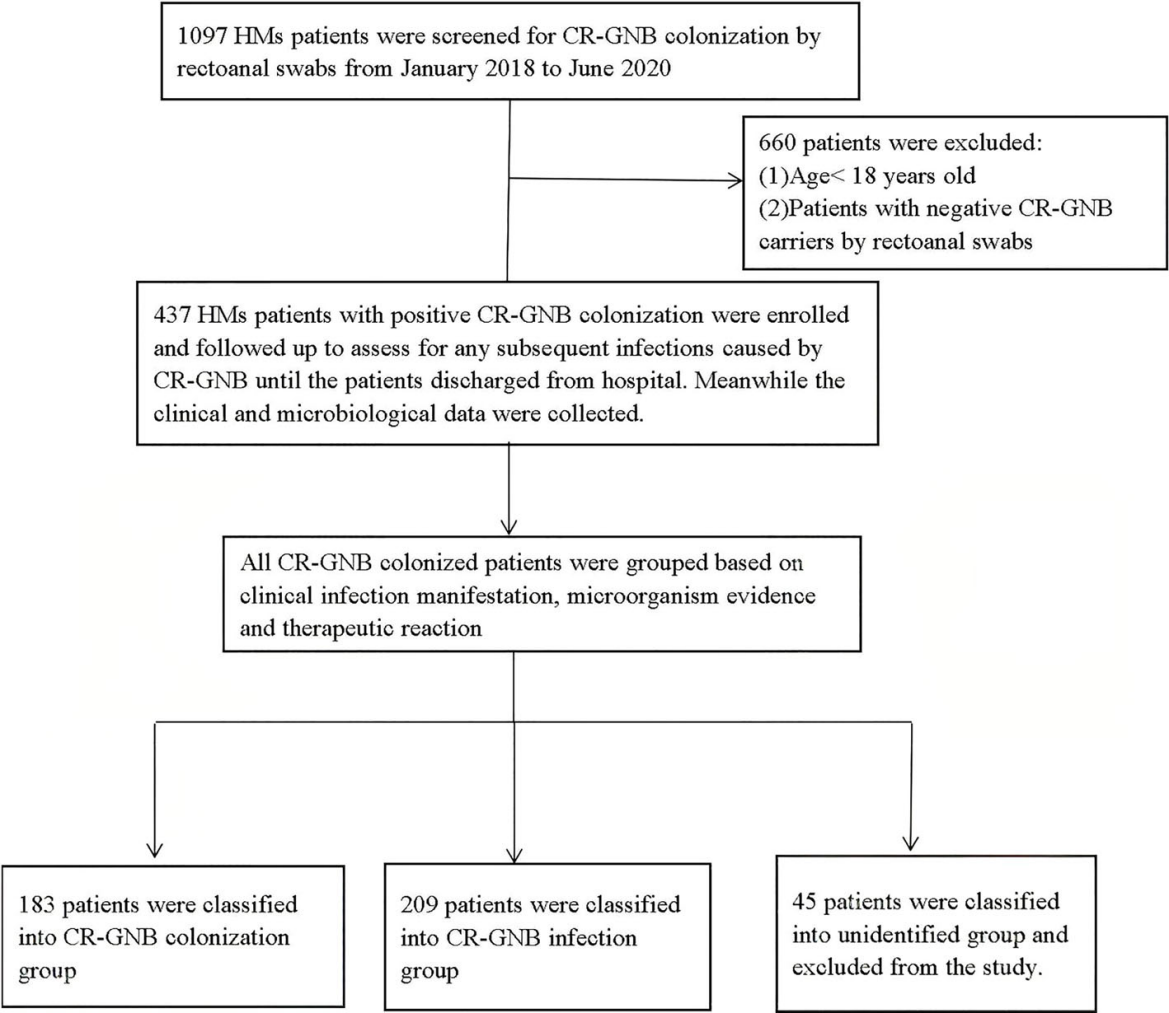
Screening algorithm of the patients with CR-GNB colonization. In all, 437 hospitalized patients had rectoanal swabs positive for CR-GNB, and a total of 392 eligible, unduplicated cases were recruited into this study.

**Figure 2:**
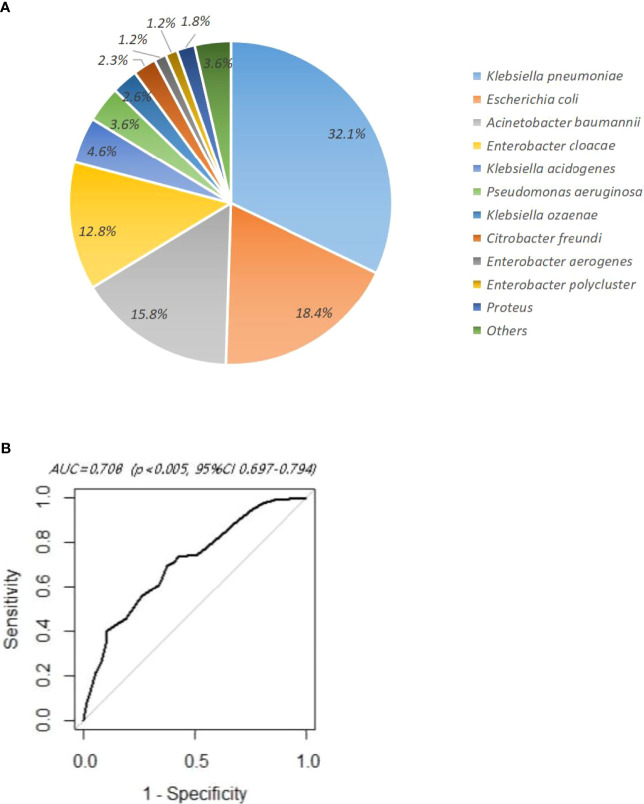
**(A)** Distribution of carbapenem-resistant Gram-negative bacteria in rectoanal swabs. **(B)** ROC curve of multivariate logistic regression analysis.

The flow procedure of this study is shown in **Figure 1**. A total of 437 HM patients with positive CR-GNB colonization were firstly included in this study, and 45 patients were classified into the unidentified group and finally excluded from our study. The data from the remaining 392 patients were further analyzed, and the top three predominant pathogens of CR-GNB colonization were *Klebsiella pneumoniae* (32.1%), *Escherichia coli* (18.4%), and *Acinetobacter baumannii* complex (15.8%). The other strains were *Enterobacter cloacae* (12.8%), *Klebsiella acidogenes* (4.6%), *Pseudomonas aeruginosa* (3.6%), *Klebsiella ozaenae* (2.6%), *Citrobacter freundi* (2.3%), *Enterobacter aerogenes* (1.2%), *Enterobacter polycluster* (1.2%), *Proteus* (1.8%), and others (3.6%) (**Figure 2A**).

### Clinical Characteristic Analysis

As shown in **Table 1**, among the 392 CR-GNB colonized patients, 183 (46.7%) subsequently developed a CR-GNB infection including 42 (10.7%) confirmed infections and 142 (36.2%) clinically diagnosed infections. The CR-GNB colonization group and infection group did not differ significantly in age and gender. In terms of disease distribution, acute myelocytic leukemia (AML) was the predominant primary disease (n=216, 55.1%), followed by acute lymphocytic leukemia (ALL) (n=69, 17.6%). CR-GNB infected patients had a longer length of hospitalization (27 d vs. 23 d, *p*=0.028) and significantly higher mortality rate (43.7% vs. 8.6%, *P* < 0.001).

**Table 1:** Basic clinical characteristics of HM patients with positive CR-GNB cultures in anal swab.

Metrics	CR-GNB colonization group (N = 209)(%)	CR-GNB infection group (N = 183) (%)	*P*	Total (%)
**Gender**				
Male	133 (63.6%)	113 (61.7%)	0.7	246 (62.8%)
Female	76 (36.4%)	70 (38.3%)		146 (37.2%)
**Age**				
<60	173 (82.8%)	162 (88.5%)	0.107	335 (85.5%)
≥60	36 (17.2%)	21 (11.5%)		57 (14.5%)
**Primary disease**				
AML	118 (56.5%)	98 (53.6%)	0.347	216 (55.1%)
ALL	31 (14.8%)	38 (20.8%)		69 (17.6%)
Lymphoma	17 (8.1%)	14 (7.7%)		31 (7.9%)
MDS	16 (7.7%)	14 (7.7%)		30 (7.6%)
CML	13 (6.2%)	4 (2.1%)		17 (4.4%)
MPAL	7 (3.3%)	7 (3.8%)		14 (3.6%)
MM	3 (1.5%)	1 (0.5%)		4 (1.0%)
Others	4 (1.9%)	7 (3.8%)		11 (2.8%)
**HSCT**				
YES	37 (17.7%)	57 (31.1%)	0.002	94 (23.9%)
NO	172 (82.3%)	126 (68.9%)		298 (76.1%)
**Survival status**				
Survival	191 (91.4%)	103 (56.3%)	<0.001	294 (75.0%)
Death	18 (8.6%)	80 (43.7%)		98 (25.0%)

HMs, hematological malignancies; AML, acute myeloblastic leukemia; ALL, acute lymphoblastic leukemia; MPAL, mixed phenotype acute leukemia; CML, chronic myeloblastic leukemia; MDS, myelodysplastic syndrome; MM, multiple myeloma; others: myeloid sarcoma(6), myelofibrosis(3), hairy cell leukemia(2).

### Risk Factors for Subsequent Infection After CR-GNB Colonization

A univariate analysis (**Table 2**) showed that the risk factors for subsequent CR-GNB infection after CR-GNB colonization included HCST, mucositis, the duration of agranulocytosis, hypoalbuminemia, pre-exposure to specific agents (carbapenem antibiotic, aminoglycoside antibiotic, remission induction chemotherapy, immunosuppressant, glucocorticoids, PPIs), and invasive procedures (CVC, sputum suction, bladder catheterization) (*p*<0.05).

**Table 2:** Risk factors of subsequent infection after CR-GNB colonization in HM patients.

Metrics		Univariate analysis			Multivariable analysis		
		Group		*P*	*P*	OR	95%CI
		CR-GNB colonization (N = 209) (%)	CR-GNB infection (N = 183) (%)				
HSCT	NO	172 (57.7%)	126 (42.3%)	0.002			
	YES	37 (39.6%)	57 (60.6%)				
Mucositis							
	No	112 (69.1%)	50 (30.9%)	<0.001	<0.001	3.248	2.089-5.114
	YES	97 (42.2%)	133 (57.8%)				
Pneumonia	NO	4 (50%)	4 (50%)	0.849			
	YES	205 (53.4%)	179 (46.6%)				
The duration of agranulocytosis							
	1-14	73 (50.3%)	72 (49.7%)	0.015	0.007	1.959	1.198-3.227
	≥15	34 (38.6%)	54 (61.4%)	<0.001	<0.001	2.651	1.509-4.717
Hypoalbuminemia							
	NO	149 (57.3%)	106 (42.7%)	0.006	0.009	1.837	1.170-2.904
	YES	60 (43.8%)	77 (56.2%)				
**Antimicrobial agent exposure**							
Cephalosporins	NO	56 (50.5%)	55 (49.5%)	0.475			
	YES	153 (54.4%)	128 (45.6%)				
Aminoglycosides	NO	139 (59.9%)	93 (40.1%)	0.002			
	YES	70 (43.8%)	90 (56.2%)				
Carbapenems	NO	95 (60.1%)	63 (39.9%)	0.026			
	YES	114 (48.7%)	120 (51.3%)				
Quinolones	NO	137 (54.2%)	116 (45.8%)	0.655			
	YES	72 (51.8%)	67 (48.2%)				
**Special drugs exposure**							
Remission induction chemotherapy							
	NO	93 (60.4%)	61 (39.6%)	0.024	0.005	1.921	1.217-3.054
	YES	116 (48.7%)	122 (51.3%)				
Immunosuppressant							
	NO	179 (56.8%)	136 (43.2%)	0.005			
	YES	30(39%)	47 (61%)				
Glucocorticoid							
	NO	112 (61.2%)	71 (38.8%)	0.003			
	YES	97 (46.4%)	112 (53.6%)				
PPIs		18 (12,26)	22 (14.75,28)	0.019			
**Invasive procedures**							
CVC	NO	58 (67.4%)	28 (32.6%)	0.003			
	YES	151 (49.3%)	155 (50.7%)				
Mechanical ventilation							
	NO	195 (54%)	166 (46%)	0.345			
	YES	14 (45.2%)	17 (54.8%)				
Others (sputum suction, bladder catheterization)							
	NO	186 (55.7%)	148 (44.3%)	0.024			
	YES	23 (39.7%)	35 (60.3%)				

HMs, hematological malignancies; HSCT, hematopoietic stem cell transplantation; PPIs, proton pump inhibitors; CVC, central venous catheter.

In order to avoid the interaction among the above risk factors and correct the bias, the factors with significant differences in univariate analysis were further included for multivariate logistic regression analysis. The results showed that remission induction chemotherapy (OR 1.921, 95%CI 1.217–3.054, *p*=0.005), the duration of agranulocytosis 1–14 days (OR 1.959, 95%CI 1.198–3.227, p=0.007), the duration of agranulocytosis ≥15 days (OR 2.651, 95%CI 1.509–4.717, *p*<0.001), mucositis (OR 3.248, 95%CI 2.089-5.114, *p*<0.001), and hypoalbuminemia (OR 1.837, 95% CI 1.170-2.904, *p*=0.009) were independent risk factors. The ROC curve showed that AUC=0.708>0.7 (*p*<0.005, 95%CI 0.697–0.794), indicating that multivariate analysis displayed an acceptable goodness of fit (**Figure 2B**).

### The Establishment of a Risk Prediction Score Model for Subsequent CR-GNB Infection After CR-GNB Colonization

In order to further quantify the proportion of independent risk factors, a scoring table was established. As shown in **Table 3**, the assignment of points based on the ORs and the regression coefficients (*βi*) for these four independent variables generated an individual risk score ranging from 0 to 6 (AUC=0.697, *p*<0.05, 95%CI: 0.647–0.747). The maximum value of the Youden index and integer up was taken as the optimal cutoff value for the scoring model. The score of independent risk factors are as follows: remission induction chemotherapy (score 1), the duration of agranulocytosis 1–14 days (score 1), the duration of agranulocytosis ≥15 days (score 2), mucositis (score 2), and hypoalbuminemia (score 1).

**Table 3:** Predictive scoring table for risk of subsequent infection after CR-GNB colonization in HM patients.

Risk factors	Category	OR	Regression coefficient *βi*	Score
Remission induction chemotherapy		1.921	0.653	
	NO			0
	YES			1
The duration of agranulocytosis				
	0			0
	1-14	1.959	0.672	1
	≥15	2.651	0.975	2
Mucositis		3.248	1.178	
	NO			0
	YES			2
Hypoalbuminemia		1.837	0.608	
	NO			0
	YES			1

According to the coordinates of the ROC curve, the optimal cutoff value was determined to be 4. Colonized patients with a total score <4 were defined as the low-risk infection group and a total score ≥4 were defined as the high-risk infection group. All colonized patients were validated in this model. Among them, 209 cases were classified into the low-risk group, and 68 cases (32.54%) had a subsequent CR-GNB infection. The rest of 183 cases were classified into the high-risk group, including 113 cases (61.75%) with a subsequent CR-GNB infection. There was a significant difference in the subsequent CR-GNB infection rates between the two groups (*p*<0.001). The OR was 3.347 (95% CI 2.218–5.094), suggesting that the risk of subsequent infection in the high-risk group was more than 3 times that in the low-risk group (**Table 4**). The sensitivity, specificity, positive predictive value, and negative predictive value were 62.4%, 67.1%, 0.6234, and 0.6682, respectively.

**Table 4:** Comparison of incidence of subsequent infection after CR-GNB colonization in different risk stratification groups.

Infection Risk Stratification	Infection rate	*P*	OR (95%CI)
Low risk (<4)	68 (32.54%)	<0.001	3.347 (2.218-5.094)
High risk (≥4)	113 (61.75%)		

## Discussion

In this retrospective study, we found that 46.7% of HM patients developed a CR-GNB infection after CR-GNB colonization, including confirmed infections (10.7%) and clinical infections (36.2%). Mucositis and the duration of agranulocytosis ≥15 days were the strongest predictors. According to our predictive scoring model including remission induction chemotherapy (score 1), the duration of agranulocytosis 1–14 days (score 1), the duration of agranulocytosis ≥15 days (score 2), mucositis (score 2), and hypoalbuminemia (score 1), the total score ≥4 suggests that HM patients with CR-GNB colonization will develop a CR-GNB infection.

The overall prevalence of CR-GNB colonization varies between 18.1% and 30.4% in different geographical regions and diseases (19–24). In our study, the CR-GNB colonization rate in HM patients was 35.7%, which was higher than that in the above study. HM patients are prone to multiple drug-resistant bacterial infections, especially during febrile neutropaenic episodes (25, 26). It has been reported that the main CR-GNB strains include *K. pneumoniae, K. acidogenes, Citrobacter freundi* in Europe, and *K. pneumoniae, Enterobacter cloacae*, and *Escherichia coli* in Asia (21, 27–34). In our study, the dominant strains were *K. pneumoniae* (32.1%), *E. coli* (18.4%), and *Acinetobacter baumanii* (15.8%), which was consistent with previous reports.

Multiple risk factors were reported to be associated with increased vulnerability for a CR-GNB infection including immunocompromise, central venous catheter, chemotherapy or radiation therapy, neutropenia, carbapenems exposure, and prior colonization (21, 22, 34, 35). Similarly, in our univariate analysis, HCST, mucositis, the duration of agranulocytosis, hypoalbuminemia, pre-exposure to specific agents (carbapenem antibiotic, aminoglycoside antibiotic, remission induction chemotherapy, immunosuppressant, glucocorticoids, PPIs), and invasive procedures (CVC, sputum suction, bladder catheterization) may be risk factors for a subsequent CR-GNB infection after CR-GNB colonization. However, in our multivariate logistic regression analysis, only remission induction chemotherapy, the duration of agranulocytosis, mucositis, and hypoalbuminemia were independent risk factors.

During remission induction chemotherapy, extremely severe immunodeficiency is most common due to both high tumor burden and potent high-dose chemotherapeutic drugs. Furthermore, the incidence of febrile granulocytopenia is highest during the first cycle of anticancer chemotherapy (36). If the patients cannot receive the remission of primary disease after induction chemotherapy, this population has a longer neutropenia duration and especially a higher risk of CR-GNB infection (37). This is in accordance with our result that remission induction chemotherapy is an independent risk factor for CR-GNB infection. In addition, transplantation patients are another population with a higher colonization prevalence due to previous chemotherapy history compared with newly diagnosed HM patients (38, 39). However, stem cell transplantation and isolation in a laminar air-flow room have been also thought to be significant factors protecting against the occurrence of Gram-negative bacterial infections (40). These can explain that transplantation was no longer a significant risk factor in the multivariate analysis in our study.

Compared with short-term agranulocytosis in patients with other malignant tumors, a long duration of agranulocytosis is more common in HM patients due to an underlying disease and high-intensity chemotherapy and leads to their significantly reduced ability to resist pathogenic microorganisms (41). Studies have shown that more than 80% of HM patients will develop infections related to neutropenia after more than 1 course of chemotherapy, compared with 10%–50% of patients with solid tumors (42). In HM patients, leukemia patients undergoing intensive induction chemotherapy have especially prolonged episodes of neutropenia. The presence of febrile neutropenia was independently associated with increased mortality in infections caused by *carbapenem-resistant Enterobacteriaceae* in HM patients in a Latin American study (43). In this cohort, we also found that the duration of agranulocytosis (≥15 days) independently increases the risk of a subsequent infection due to CR-GNB colonization and thus negatively affects their clinical course.

Mucositis is a serious and debilitating side effect of cytotoxic chemotherapy and persistent reduction of neutrophils (44, 45). Herein, we found that mucositis is another independent risk factor for a CR-GNB infection. Oral and gut microbiome alterations are prevalent in HM patients due to the administration of chemotherapeutic drugs and broad-spectrum antibiotics, which favor the colonization or excessive growth of CR- GNB. Oral and gastrointestinal mucositis result in the damage of the mucous membrane barrier and promote colonized CR-GNB to enter into the blood circulation (46, 47). In HM patients with mucositis, CR-GNB-colonized patients may require increased vigilance for sepsis detection, owing to an increased risk for gut translocation and endogenous infection development.

Hypoalbuminemia is a common complication in hematological malignancies due to inadequate nutrition intake and cachexia, which are correlated with increased vascular permeability and interstitial volume. Furthermore, serum albumin has the effects of anti- oxidation and anti-apoptosis. Its reduction will cause low host immunity, a delayed repair of microcirculatory mucosal injury, and increased infection (48). A retrospective chart review confirmed that hypoalbuminemia is a clinical predictor of early infection in HM patients (49). In this study, a multivariate logistic showed that hypoalbuminemia was an independent risk factor for subsequent CR-GNB infection after CR-GNB colonization and accounted for 1 point in our predictive scoring model.

The prediction score model of infection for HM patients with CR-GNB colonization was seldomly reported. Recently, a risk prediction model for CRE bloodstream infection (BSI) in intestinal carriers in the hematology department and intensive care unit (ICU) was established in a retrospective study (50). Gastrointestinal injury, tigecycline exposure, and carbapenem resistance score were chosen as valuable markers for the risk prediction model of CRE BSI in intestinal carriers. However, the BSI rates are very low for HM patients (4.7%~23.1% in Europe and America and 4.6%-8.9% in China) (21, 51, 52), these prediction score models may miss some clinically infected patients with negative blood culture. Therefore, in this study, the patients were grouped by combining clinical infection manifestation, the microorganism evidence, and therapeutic reaction to antibiotics. Both CR-GNB microbiologically documented infections and clinical CR-GNB infections were included in CR-GNB infection group; only 10.2% of patients had positive blood cultures. We selected four independent variables including remission induction chemotherapy (score 1), the duration of neutropenia (score 1 for 1–14 days and score 2 for ≥15 days), mucositis (score 2), and hypoalbuminemia (score 1) to establish our predicting model and divided all the colonized patients into a low-risk group (<4 points) and a high-risk group (≥4 points). Our model has a certain sensitivity and specificity and is in high accordance with subsequent CR-GNB infection rates, and the high-risk group was >3 times more likely to develop a subsequent infection in comparison with the low-risk group (OR3.347, 95%CI 2.218–5.094, p<0.001). Therefore, the early administration of CR-GNB-targeted empirical therapy in a high-risk group is strongly recommended to decrease their mortality. Meanwhile, for those patients during low-risk periods, anti-non-CR-GNB treatment and close monitoring might be enough.

This study has several important limitations including its retrospective, observational design. Due to species preservation and equipment, we did not identify the enzyme type of CR-GNB strain and there is no information on the specific carbapenemases (KPC, OXA, etc.) identified in our studied population. An external validation cohort is needed to assess its discriminatory ability and goodness of fit. Additionally, the sample size of the transplantation group was still relatively small, limiting our ability to conduct a statistically significant comparison between the transplantation group and the nontransplantation group. Finally, it is needed to determine whether the established scoring model is reproducible through relevant prospective studies.

In conclusion, a CRGNB infection has become a major threat to public health, especially in HM patients. Not all CR-GNB colonized patients subsequently develop a CR-GNB clinical infection. Therefore, our model is of great clinical value to guide the clinicians for the selection of early prophylactic anti-CR-GNB treatment to reduce mortality during high-risk periods. Meanwhile for those patients during low-risk periods, anti-non-CR-GNB treatment and close monitoring might be enough, which can decrease the mortality rate, bacterial resistance rate, and healthcare costs.

## Data Availability Statement

The raw data supporting the conclusions of this article will be made available by the authors, without undue reservation.

## Ethics Statement

The studies involving human participants were reviewed and approved by Independent Ethics Committee of Union Hospital, Tongji Medical College, Huazhong University of Science and Technology. Written informed consent for participation was not required for this study in accordance with the national legislation and the institutional requirements.

## Author Contributions

MH conceived and designed the study. QW, CQ, HY, FL, YW, WL, LX, and LM collected and analyzed data. QW and CQ wrote the paper. MH, LX, and LM reviewed and edited the manuscript. All authors read and approved the manuscript.

## Funding

This study was supported by the National Natural Science Foundation of PR China (no. 81570193 and no. 81770219, for QW).

## Conflict of Interest

The authors declare that the research was conducted in the absence of any commercial or financial relationships that could be construed as a potential conflict of interest.

## Publisher’s Note

All claims expressed in this article are solely those of the authors and do not necessarily represent those of their affiliated organizations, or those of the publisher, the editors and the reviewers. Any product that may be evaluated in this article, or claim that may be made by its manufacturer, is not guaranteed or endorsed by the publisher.
